# Exploring Motivations for COVID-19 Vaccination Among Black Young Adults in 3 Southern US States: Cross-sectional Study

**DOI:** 10.2196/39144

**Published:** 2022-09-02

**Authors:** Marie CD Stoner, Erica N Browne, David Tweedy, Audrey E Pettifor, Allysha C Maragh-Bass, Christina Toval, Elizabeth E Tolley, Maria Leonora G Comello, Kathryn E Muessig, Henna Budhwani, Lisa B Hightow-Weidman

**Affiliations:** 1 RTI International Berkeley, CA United States; 2 University of North Carolina Chapel Hill, NC United States; 3 FHI 360 Durham, NC United States; 4 Florida State University Tallahassee, FL United States

**Keywords:** COVID-19, COVID-19 vaccination, young people, vaccination motivations, vaccination beliefs, online survey, health disparity, minority population, vaccine hesitancy, misinformation, vaccine safety

## Abstract

**Background:**

Few studies have focused on attitudes toward COVID-19 vaccination among Black or African American young adults (BYA) in the Southern United States, despite high levels of infection in this population.

**Objective:**

To understand this gap, we conducted an online survey to explore beliefs and experiences related to COVID-19 vaccination among BYA (aged 18-29 years) in 3 southern states.

**Methods:**

We recruited 150 BYA to participate in an online survey as formative research for an intervention to address vaccine hesitancy in Alabama, Georgia, and North Carolina from September 22, 2021, to November 18, 2021. Participants were recruited through social media ads on Facebook, Twitter, Instagram, and YouTube. Additionally, we distributed information about the survey through organizations working with BYA in Alabama, Georgia, and North Carolina; our community partners; and network collaborations. We used measures that had been used and were previously validated in prior surveys, adapting them to the context of this study.

**Results:**

Roughly 28 (19%) of the participants had not received any doses of the COVID-19 vaccine. Half of the unvaccinated respondents (n=14, 50%) reported they wanted to wait longer before getting vaccinated. Motivators to get vaccinated were similar between unvaccinated and vaccinated respondents (eg, if required, to protect the health of others), but the main motivator for those vaccinated was to protect one’s own health. Among unvaccinated individuals, reasons for not receiving the COVID-19 vaccine included concern about vaccine side effects (n=15, 54%) and mistrust of vaccine safety (n=13, 46%), of effectiveness (n=12, 43%), and of the government’s involvement with vaccines (n=12, 43%). Experiences of discrimination (n=60, 40%) and mistrust of vaccines (n=54, 36%) were common overall. Among all respondents, those who said they would be motivated to get vaccinated if it was required for school, work, or travel were more likely to endorse negative beliefs about vaccines compared to those motivated for other reasons.

**Conclusions:**

Mistrust in COVID-19 vaccine safety and efficacy is common among BYA in the Southern United States, irrespective of vaccination status. Other motivators, such as safety of family and community and vaccination requirements, may be able to tip the scales toward a decision to be vaccinated among those who are initially hesitant. However, it is unclear how vaccine requirements among BYA in the South affect trust in the government or health care in the long term. Interventions that include BYA in vaccination messaging and programs may more proactively build feelings of trust and combat misinformation.

## Introduction

Young adults (18-29 years) are a priority population among which to increase COVID-19 vaccine uptake because they have high rates of asymptomatic infection and transmission [1-3]. For example, a study of 767 hotspot counties early in the pandemic in 2020 reported that percentage positivity increased earliest in younger persons (aged 18-24 years), followed by several weeks of increasing percentage positivity among older age groups, particularly in hot spots within the Southern and Western United States [1]. These findings corroborate patterns seen in another study from the Southern United States, where increased percentage positivity among young adults preceded increases among those aged ≥60 years by 4-15 days [4]. Given that many young adults live in multigenerational households with older loves ones, they are also potentially a source of COVID-19 transmission to older adults, who are more likely to be vulnerable to COVID-19 irrespective of race [5]. Roughly, a third of young adults lived in multigenerational households in 2021, and Black or African American young adults (BYA) were more likely than White young adults to live in these multigenerational households [6]. Although young people may be more likely to transmit COVID-19 than other demographics, they are also potential change agents to promote safer behaviors within their families and communities. Young adults who identify as Black or African American (referred to as Black throughout this paper) have critical social capital to influence health behaviors in others, including in adult family and community members who are at high risk for COVID-19 hospitalization and mortality [7]. Therefore, BYA should be prioritized in efforts to increase COVID-19 vaccination because they make an important contribution to sustaining community transmission and can serve as change agents in their communities.

Multiple vaccines have been proven to be efficacious in preventing hospitalization and severe COVID-19 infection and are now widely available [8,9]. Despite the high efficacy, the rapid development and approval of these vaccines have resulted in public concern around vaccine safety, and many remain hesitant toward vaccination [10]. Concern around vaccines is particularly salient in Black communities with deeply rooted medical mistrust due, in part, to well-known ethical violations, such as Henrietta Lacks and the Tuskegee study and structures, policies, and practices rooted in structural racism that create health inequities in many Black communities [10-14]. Several studies have identified that vaccine hesitancy varies by age and race, with Black communities having higher rates of vaccine hesitancy and younger age being associated with greater hesitancy across race/ethnicity [10,15-17]. However, few studies focus specifically on BYA in the Southern United States who are an important population among which to increase vaccination, including both completion of the primary vaccine series and receipt of booster shots. As of March 28, 2022, the percentage with at least 1 dose of the vaccine was 65% in Georgia, 62% in Alabama, and 83% in North Carolina, lower in 2 of the 3 states than the US national average of 77% [18]. We conducted an online survey with BYA (aged 18-29 years) in Georgia, Alabama, and North Carolina to describe rates of COVID-19 vaccination acceptance and known correlates (their experiences with health care, beliefs around vaccines in general and with the COVID-19 vaccine, and demographic factors). This survey was designed as a formative assessment to inform content for a digital health intervention to address COVID-19 vaccination hesitancy among BYA in these states.

## Methods

### Study Population and Recruitment

We recruited 150 BYA to participate in an online survey related to COVID-19 vaccination in Alabama, Georgia, and North Carolina in the Southern United States (Budhwani et al, unpublished data, 2022). Eligibility criteria for the online survey included (1) 18-29 years of age, (2) identified as African American or Black, (3) English proficient, (4) access to a personal smartphone, and (5) resident of Georgia, Alabama, or North Carolina. We aimed to recruit an approximately equal number of respondents from each state: 44 (29%) respondents from Alabama, 50 (33%) from Georgia, and 56 (38%) from North Carolina. Participants completed a 1-time online survey through the Qualtrics survey platform.

Participants were recruited through social media ads on Facebook, Twitter, Instagram, and YouTube. Additionally, we distributed information about the survey through organizations in the study areas working with BYA, our community partners, and other network collaborations. BYA interested in participating in the study clicked a link within an email notification or via social media and were redirected to a study website, where they completed eligibility screening. All potential participants completed an online screening survey via Qualtrics to obtain consent to be screened and to verify inclusion criteria. The online screening survey was about 5 minutes in length and included a script that was read by the participants that explained the purpose of screening and the general purpose of the study and clarified that if they were eligible, they would be invited to participate in the study. We used established stringent verification procedures utilized in multiple prior studies to address fraud (eg, assessment for duplicate entries and suspicious response patterns, confirmation of the internet protocol [IP] address, zip code, and latitude and longitude) [19-21]. Eligible BYA were then directed to an informed consent page. Participants agreed to participate by clicking forward and providing online electronic consent (e-consent) or declined by exiting the website. The survey took approximately 30-45 minutes to complete. All respondents received a US $20 incentive for participation and the ability to participate in a lottery, where they were eligible to receive a larger prize.

The survey was conducted from September to November 2021. At this time during the COVID-19 pandemic, vaccines were available for all respondents who were aged 18-29 years. The Food and Drug Administration (FDA) issued an Emergency Use Authorization (EUA) for the Pfizer-BioNTech COVID-19 vaccine on December 11, 2020; for the Moderna vaccine on December 28, 2020; and for the Janssen (Johnson & Johnson) COVID-19 vaccine on February 27, 2021 [22]. Before the study period and on September 22, 2021, the FDA authorized a booster dose of the Pfizer-BioNTech COVID-19 vaccine for individuals aged 65 years and older, aged 18-64 years at high risk of severe COVID-19, and aged 18-64 years who had institutional or occupational exposure to SARS-CoV-2 [22]. On November 29, 2021, just after the survey period, the Centers for Disease Control and Prevention (CDC) expanded its recommendation that all individuals aged 18 years and older get a COVID-19 booster [22].

### Ethical Considerations

The study was overseen by the University of North Carolina (UNC), Chapel Hill (Approval #21-1746), with a reliance agreement from the Institutional Review Boards at the University of Alabama, RTI International, and FHI 360.

### Measures

Survey measures were selected according to constructs of social cognitive theory (SCT) to understand behavioral, cognitive (eg, knowledge), and environmental (eg, social influences by parents, peers, and community) influences motivating COVID-19 vaccination. We used measures that had been used and were previously validated in prior surveys, adapting them to the context of this study. To make these domains and constructs more specific to COVID-19 vaccination, we referred to the National Institute on Minority Health and Health Disparities (NIMHD) research framework, which is informed by the World Health Organization (WHO) Strategic Advisory Group of Experts (SAGE) [23]. We adapted the NIMHD framework to reflect determinants of vaccine hesitancy for BYA within different levels of influence (eg, individual, interpersonal, community) [24]. We examined individual, interpersonal, and community influences on vaccination as these different sources of social influence have all been associated with health behaviors in young adults. Vaccine beliefs were measured using 10 items on a 5-point Likert scale adapted from a validated scale developed by Shapiro et al [25]. Measures of vaccine confidence were based on the CDC COVID-19 Vaccine Confidence Rapid Community Assessment Tool, which is validated and has been previously used [26]. Questions related to reasons and motivations for vaccination were adapted from the RADX-up Common Data Elements (CDEs) set of validated measures [27]. When asked about their motivations, respondents were asked to select their main motivation for being or not being vaccinated from a preset list and to choose all that apply [27].

### Statistical Analysis

We descriptively examined characteristics, including participant demographics and knowledge, attitudes, and beliefs regarding vaccination, in general and about the COVID-19 vaccine, as well as prevention practices in the overall sample. We used frequencies and percentages for categorical variables and medians with the IQR for continuous variables. We tested for difference in vaccination by state using a chi-square test. We also examined motivations and barriers among those fully vaccinated (2 doses of a 2-dose vaccine, or 1 dose of a 1-dose vaccination) versus those not vaccinated and grouped vaccine hesitancy characteristics into their corresponding SCT domains/constructs to assess how these components influence vaccination behaviors. Although some measures were items from scales, all items were examined individually rather than as a score in order to understand answers to specific responses. The full survey is available upon request. In the results, we focus on environmental determinants of vaccination, which were a large contributor to overall attitudes about vaccination.

## Results

### Demographic Characteristics

Among 150 BYA who completed the survey between from September to November 2021, the median age was 23 (IQR 20-26) years, 88 (59%) identified as cisgender women, 41 (27%) as cisgender men, 4 (3%) as transgender, and 18 (12%) as nonbinary, genderqueer, gender nonconforming, or gender fluid (Table 1). One-third were employed as essential workers (n=49, 33%), 44 (29%) lived in a household with any children under 18 years old, and 61 (61%) either had a chronic condition themselves or currently lived with someone with a chronic health condition. Most respondents (n=123, 82%) reported ever having at least 1 negative experience with a health care provider, where a provider did not believe they were telling the truth, assumed something without asking, did not listen to them, or suggested they were personally to blame for a health problem. Half (n=69, 49%) thought they were treated this way specifically because of their race.

Approximately 112 (75%) of the respondents had received 2 doses of a messenger RNA (mRNA) vaccine or 1 dose of the Johnson & Johnson COVID-19 vaccine, 10 (7%) received 1 dose of a 2-dose vaccine, and 28 (19%) had not received a vaccine. Vaccination was similar across states (n=49, 87% in North Carolina; n=38, 76% in Georgia; n=35, 80% in Alabama; *P*=.30). Among those who were fully vaccinated, less than half (n=71, 47%) planned to get a booster shot; however, this was before the CDC strengthened its recommendation on November 29, 2021, for all individuals over 18 years old to receive the booster [22]. Less than 15% had ever been diagnosed with COVID-19 (n=18, 12%), but 79 (53%) knew someone who had been hospitalized or died as a result of having COVID-19.

**Table 1:** Demographics of 150 BYA^a^ who participated in the survey (September-November 2021).

Characteristics		Participants
Age (years), median (IQR)		23 (20-26)
Female sex, n (%)		103 (69)
**Gender identity, n (%)**		
	Cisgender woman	88 (59)
	Cisgender man	41 (27)
	Transgender	4 (3)
	Nonbinary, genderqueer, gender nonconforming, or gender fluid	18 (12)
**Sexual orientation, n (%)**		
	Gay or lesbian	10 (7)
	Straight	87 (58)
	Bisexual	35 (23)
	Queer, pansexual, asexual, or other	28 (19)
**Highest level of education completed, n (%)**		
	Less than high school	2 (1)
	High school	103 (69)
	Bachelor's degree or higher	45 (30)
Employed as an essential worker, n (%)		49 (33)
Have health insurance, n (%)		106 (71)
Household size, median (IQR)		2 (1-4)
Children <18 years old in the household, n (%)		44 (29)
Self or household member with a chronic health condition, n (%)		91 (61)
Smoke tobacco, n (%)		22 (15)
**State of residence, n (%)**		
	Alabama	44 (29)
	Georgia	50 (33)
	North Carolina	56 (37)
**COVID-19 vaccination and diagnosis status, n (%)**		
	Received both doses or one dose of the Johnson & Johnson	112 (75)
	Received one dose of a two-dose vaccine, intend to get second dose	10 (7)
	Not received the vaccine	28 (19)
	Planning to get a booster	71 (47)
	Ever diagnosed with COVID-19	18 (12)

^a^BYA: Black or African American young adults.

### Motivations for Vaccination

Of those BYA who were fully vaccinated (n=122, 81%), the main reason for accepting the vaccine was to protect their own health (n=56, 46%); to protect the health of family/friends, coworkers, or community members (n=33, 27%); and to resume work, school, social activities, or travel (n=26, 21%); see Table 2. When able to choose more than 1 motivation, the mean number of reasons selected for receiving the COVID-19 vaccine was 4.1 (range 1-8). About half (n=56, 46%) reported that most or all family members had been vaccinated, 69 (57%) reported that most or all close friends had been vaccinated, and 87 (71%) reported that community leaders supported vaccination. Most were confident that the COVID-19 vaccines are safe (n=109, 85%) and effective at preventing severe illness (n=109, 89%), but 37 (30%) had heard information about the COVID-19 vaccine that they could not determine was true. In addition, 34 fully vaccinated respondents (28%) had little to no trust in the government and public health agencies’ vaccine recommendations.

Among those who were unvaccinated (n=28, 19%), the main motivators to get vaccinated were if it was required for school, work, or travel (n=10, 36%), to protect the health of family/friends (n=7, 25%), and to protect one’s own health (n=3, 11%); 4 (14%) participants did not know what would motivate them (Table 3). Less than a third were confident that the COVID-19 vaccines are safe (n=8, 29%) and effective (n=6, 21%). The main reasons unvaccinated BYA noted not receiving the COVID-19 vaccine included concern about vaccine side effects (n=6, 21%), not trusting that the vaccine is safe (n=7, 25%), not knowing enough about how well a COVID-19 vaccine works (n=4, 14%), waiting until more people have gotten it (n=4, 14%), and not trusting the government (n=3, 11%). When able to choose more than 1 main motivator, the mean number of reasons selected for not receiving the COVID-19 vaccine was 2.3 (range 0-6). Half of the unvaccinated sample (n=14, 50%) reported wanting to wait to get vaccinated until it had been available for a while to see how it is working for other people; 4 (14%) participants said they would definitely not get the vaccine.

**Table 2:** Motivations for receiving the COVID-19 vaccine.

Motivations			Participants (N=122)
**Main reason chose to get the COVID-19 vaccine, n (%)**			
	To protect my health	56 (46)	
	To protect the health of family/friends, coworkers or my community	33 (27)	
	To resume work, school, social activities, or travel	26 (21)	
	Because others I trust encouraged me to get it	5 (4)	
	Other	2 (3)	
Mean number of reasons selected for COVID-19 vaccination when able to choose more than 1, mean (range)			4.1 (1-8)

**Table 3:** Reasons for not getting the COVID-19 vaccine.

Reasons			Participants (N=28)
**Main reason for not getting the COVID-19 vaccine, n (%)**			
	Do not trust that the vaccine is safe	7 (25)	
	Concerned about side effects from the vaccine	6 (21)	
	Waiting until more people have gotten it	4 (14)	
	Do not know enough about how well a COVID-19 vaccine works	4 (14)	
	Do not trust the government	3 (11)	
	Just have not gotten around to getting it	2 (7)	
	Other	2 (7)	
Mean number of reasons selected for not getting COVID-19 vaccination when able to choose more than 1, mean (range)			2.3 (0-6)
**Main reason that would motivate to get the COVID-19 vaccine, n (%)**			
	To protect my health	3 (11)	
	To protect the health of family/friends, coworkers, or community	7 (25)	
	To resume work, school, social activities, or travel	10 (36)	
	Because others I trust encouraged me to get it	1 (4)	
	Other	3 (11)	
	Do not know	4 (14)	

### Attitudes Toward Vaccines

Overall, nearly all respondents (n=138, 92%) believed that, in general, vaccines are useful and effective, and many (n=90, 60%) reported prior lifetime receipt of the flu vaccine (Table 4). However, there was a high level of overall mistrust in vaccines; the participants believed that people are deceived about how well vaccines work to prevent illness (n=54, 36%) and about vaccine safety (n=55, 37%). Approximately one-third thought pharmaceutical companies cover up the dangers of vaccines (n=47, 31%) and that the government is experimenting with vaccines on the Black community (n=43, 29%); see Table 4.

Vaccine beliefs differed by the underlying motivation to receive the COVID-19 vaccine. Those who said they were or would be motivated to get vaccinated if it was required for school, work, or travel were more likely to endorse all negative beliefs about vaccination compared to those motivated for other reasons (Table 5). For example, 21 (58%) of those motivated by vaccine requirements agreed that the government is experimenting with vaccines on the Black community compared to 10 (25%) of those motivated to protect health of others and 11 (19%) of those motivated to protect their own health. Most (n=21, 58%) of those motivated by vaccine requirements agreed that people are deceived about vaccine effectiveness compared to 9 (23%) of those motivated to protect health of others and 15 (25%) of those motivated to protect their own health.

**Table 4:** Beliefs about vaccination and experiences with health care.

Beliefs and experiences		Participants (N=150), n (%)
**Social network and COVID-19**		
	Know anyone who has been vaccinated	133 (89)
	Most or all family members vaccinated	61 (41)
	Most or all close friends vaccinated	76 (51)
	Leaders (religious, political, teachers, health care workers) in your community support getting vaccinated	103 (69)
	Know someone personally who has been hospitalized or died because of COVID-19	79 (53)
**COVID-19 vaccine confidence**		
	Confident currently available COVID-19 vaccines in the United States are effective at preventing severe illness sick or hospitalization	115 (77)
	Confident COVID-19 vaccines currently available in the United States are safe	112 (75)
	Know where to get accurate, timely information about COVID-19 vaccines	110 (73)
	I have heard information about COVID-19 vaccines that I cannot determine is true or false	50 (33)
	Trust the government and public health agencies that recommend getting a COVID-19 vaccine	94 (62)
**Negative experiences with health care: Have you ever felt that a doctor or health care provider:**		
	Did not believe you were telling the truth?	81 (54)
	Refused to order a test or treatment you thought you needed?	69 (46)
	Suggested you were personally to blame for a health problem you were experiencing?	67 (45)
	Assumed something about you without asking?	83 (55)
	Talked down to you or did not treat you with respect?	73 (49)
	Did not listen to what you had to say?	81(54)
	Ever experience racial discrimination from health care provider?	60 (40)
**Vaccine beliefs (agree or strongly agree)**		
	Vaccine safety data are often fabricated.	33 (22)
	Pharmaceutical (drug) companies cover up the dangers of vaccines.	47 (31)
	People are deceived about how well vaccines work to prevent illness and death.	54 (36)
	People are deceived about vaccine safety.	55 (37)
	The government covers up the link between vaccines and autism.	22 (15)
	The government is experimenting with vaccines on the Black community.	43 (29)
	Vaccines have many known harmful side effects.	50 (33)
	Vaccines may lead to illness and death.	43 (29)
	Vaccines provide important benefits to society.	133 (89)
	Vaccines are useful and effective.	138 (92)
**Vaccine experience**		
	Ever received the flu vaccine	90 (60)
	Received the flu vaccine in the past 12 months	44 (29)

**Table 5:** Vaccine beliefs by motivations for receiving the COVID-19 vaccine.

Vaccine beliefs			COVID-19 vaccination motivation, n (%)								
		To protect my health		To protect the health of others		Required		Other or do not know^a^		Total	
Total			59 (100)		40 (100)		36 (100)		15 (100)		150 (100)
**Vaccine safety data are often fabricated.**											
	Agree	8 (14)		7(18)		12 (33)		6 (40)		33 (22)	
	Disagree or do not know	51 (86)		33 (83)		24 (67)		9 (60)		117 (78)	
**Pharmaceutical (drug) companies cover up the dangers of vaccines.**											
	Agree	14 (24)		6 (15)		17 (47)		10 (67)		47 (31)	
	Disagree or do not know	45 (76)		34 (85)		19 (53)		5 (33)		103 (69)	
**People are deceived about how well vaccines work to prevent illness and death.**											
	Agree	15 (25)		9 (23)		21 (58)		9 (60)		54 (36)	
	Disagree or do not know	44 (75)		31 (78)		15 (42)		6 (40)		96 (64)	
**People are deceived about vaccine safety.**											
	Agree	18 (31)		8 (20)		20 (56)		9 (60)		55 (37)	
	Disagree or do not know	41 (70)		32 (80)		16 (44)		6 (40)		95 (63)	
**The government covers up the link between vaccines and autism.**											
	Agree	5 (9)		5 (13)		6 (17)		6 (40)		22 (15)	
	Disagree or do not know	54 (92)		35 (88)		30 (83)		9 (60)		128 (85)	
**The government is experimenting with vaccines on the Black community.**											
	Agree	11 (19)		10 (25)		17 (47)		5 (33)		43 (29)	
	Disagree or do not know	48 (81)		30 (75)		19 (53)		10 (67)		107 (71)	

^a“^Because others I trust encouraged me to get it” (n=6, 40%), “other” (n=5, 33%), and “do not know” (n=4, 27%).

## Discussion

### Principal Findings

We conducted an online survey to describe COVID-19 vaccine acceptance and correlates among young Black adults in 3 southern states experiencing COVID-19 inequities. We found that approximately 14% of respondents had not received any doses of the COVID-19 vaccine as of November 2021. Most unvaccinated respondents reported that they were waiting to get vaccinated until it had been available for a while. Both vaccinated and unvaccinated respondents described similar motivations for vaccination, including to protect their own health, to protect the health of their family and communities, and requirements to resume work, school, travel, or social activities. Mistrust of vaccines was common even among the vaccinated population and was a barrier among the unvaccinated. Among all respondents, those who said they were or would be motivated to get vaccinated if it was required for school, work, or travel were more likely to endorse negative beliefs about vaccination compared to those motivated for health reasons. Overall, our findings show that strategies to increase vaccination among BYA should emphasize the health of the family and community, while also continuing to build trust in vaccination overall and specifically around the safety and efficacy of the COVID-19 vaccine. Strategies that use vaccination mandates should consider and address how these requirements may also affect trust in vaccination. These findings are being used to inform the content for a smartphone application to address vaccination BYA in Georgia, Alabama, and North Carolina [24] (Budhwani et al, unpublished data, 2022).

### Comparison With Prior Work

Despite the relatively young age of our sample, experiences of discrimination by a health care provider and mistrust of vaccines and the government’s involvement with vaccines were common. Among those unvaccinated, the main concerns about receiving the COVID-19 vaccine were side effects, trust of the government, trust in vaccines in general and specifically of the safety and efficacy of the COVID-19 vaccine, and not having enough information about how well the COVID-19 vaccine works. Our findings are similar to a systematic review of 13 studies that found that major predictors of vaccine hesitancy in Black and Hispanic people were medical mistrust and a history of racial discrimination; exposure to myths and misinformation; beliefs about vaccines and past vaccine compliance; and concerns about the safety, efficacy, and side effects of the COVID-19 vaccines [10]. Concerns about safety and efficacy have been shown to be a major barrier to vaccination across populations and countries [28]. Additional studies among college populations have also shown that a common reason for unwillingness to receive the vaccine are the belief that the vaccine approval process was rushed [15], as well as mistrust of the health care system or government held by Black individuals, as described earlier [29]. Our findings indicate that mistrust was also common among vaccinated BYA, suggesting the potential for other motivations to build trust and facilitate vaccine uptake, such as trusted sources to combat misinformation and inclusion of BYA in vaccination messaging and programs, given their potential as change agents.

A review of interventions to increase vaccination for any indication found that aside from provider recommendations, the most effective interventions to increase vaccination have been those that target direct behavior change through the use of policies and practices to increase vaccination (eg, mandates, incentives, transportation) without changing what people think or feel (eg, vaccine confidence or perceived risk) [30-32]. Evidence from randomized trials has also shown that interventions to build on intentions (eg, reminders, prompts, and reducing logistical barriers) and shape behavior (eg, incentives, sanctions, or requirements, such as work and school vaccination mandates) have been effective at increasing vaccination [30-32]. In our survey, we found that many respondents were waiting to eventually receive the vaccine and might benefit from strategies to facilitate vaccination, although vaccines had already been approved for almost a year at the time of the survey [22]. In addition, we found that almost a third of those who were vaccinated and unvaccinated reported that their main motivation for vaccination was to resume work, school, travel, or other social activities. Yet, those who said they were or would be motivated because of vaccine requirements were more likely to endorse negative beliefs about vaccination compared to those motivated by other reasons. Although requirements may be an important strategy and can result in greater vaccine uptake, it still leaves a gap in building trust in vaccines and does nothing to address or may reinforce justified medical skepticism within the Black community. Effective intervention strategies to address mistrust have included communicating the importance of vaccination through trusted channels (eg, Black physicians and clinicians), validating the real history- and experience-based reasons people may be hesitant, and addressing racism embedded within the health care system [33]. Interventions are critically needed to build trust and address medical mistrust, including through meaningful involvement of BYA in polices and programming for this age group.

Medical mistrust and a history of abuse and racism from the medical and research establishment mean that increasing vaccine uptake and trust in the safety and efficacy of COVID-19 vaccines requires messaging with involvement from trusted sources in the Black community and that the role of social support in encouraging vaccination is key. Our findings largely resonate with those of a prior study by Balasuriya et al [34] that used qualitative interviews with 72 respondents to explore COVID-19 vaccine access and acceptance among Black and Latinx communities in March 2021. The study identified 3 major themes that represent facilitators of and barriers to COVID-19 vaccination: (1) pervasive mistreatment of Black and Latinx communities and associated distrust; (2) informing trust via trusted messengers and messages, choice, social support, and diversity; and (3) addressing structural barriers to vaccination access. The first theme is related to pervasive mistreatment of Black and Latinx communities both historically and throughout the pandemic and aligns with the high levels of mistrust that were observed in our sample and high reported experiences of discrimination in health care. Within the second theme, respondents described feelings of trust in the COVID-19 vaccine that were built through receiving information from trusted messengers, consistent and transparent messaging, social support, and seeing diversity at the vaccination site. Accordingly, our results also show that despite mistrust even among vaccinated individuals, family and community concern was a motivator to receive the vaccine. Additionally, questions and skepticism about vaccination can be protective and efforts are needed to ensure that institutions are trustworthy, transparent, and engaged with communities throughout the vaccine rollout and other public health efforts [35].

### Limitations

Our study used a convenience sample of young adults from North Carolina, Georgia, and Alabama and may not be representative of the larger population of BYA in these states. We observed that 86% of our sample was vaccinated, a rate much higher than national- and state-level data [36,37]. As of November 1, 2021, 59.3% of adults over the age of 18 years were vaccinated in Georgia, 54.8% in Alabama, and 63.4% in North Carolina [37]. The higher percentage vaccinated in our study may be because unvaccinated BYA are less likely to want to participate in research or because the survey was not distributed as widely through the social networks of unvaccinated individuals. There was also an overrepresentation of those assigned the female sex at birth (69%) in the sample. In addition, our sample included only 150 BYA, with 28 unvaccinated individuals. Given the small sample size and biased sample, we had limited power and ability to make statements about this population or specific comparisons between those vaccinated or unvaccinated. Lastly, the science and evidence have been evolving quickly on COVID-19, and this survey was conducted in September-November 2021, before the CDC expanded its recommendation on November 29, 2021, that all individuals ages 18 years and older get a COVID-19 booster [22]. Therefore, we are unable to make conclusions about boosters in this population, although more recent evidence suggests the percentage of those who have received a booster shot is lower in those aged 18-29 years compared to other age groups [36]. Future studies are needed to determine strategies to increase booster shots, particularly as concerns about vaccination were common among both vaccinated and unvaccinated groups in our survey and may lead to a lower likelihood of boosters. We also did not ask about COVID-19 testing since 2020. Therefore, the percentage who were ever diagnosed with COVID-19 (12%) is likely an underestimate of the percentage of the population who had truly been infected.

### Conclusion

In a convenience sample of 150 BYA (aged 18-29 years) in Georgia, Alabama, and North Carolina, most respondents had received the COVID-19 vaccine or reported wanting to wait to get vaccinated until it had been available for a while to see how it is working for other people. Reporting of mistrust of vaccines, particularly of vaccine safety and efficacy, was common and was noted as a barrier to receiving a vaccine among those who were unvaccinated and was also pervasive among those who were vaccinated. Protecting the health of the family and community, protecting individual health, and vaccine requirements were motivators for vaccination among both vaccinated and unvaccinated individuals. Yet, those who said they were or would be motivated because of vaccine requirements were more likely to endorse negative beliefs about vaccination compared to those motivated by other reasons. Strategies such as vaccine requirements may be able to tip the scales toward a decision to get vaccinated among those who are initially hesitant—at least in the short term. However, it is unclear how vaccine requirements among BYA in the South will affect trust in the government or health care in the long term. Our study suggests that whether vaccine accepting or hesitant, interventions are critically needed to build trust in vaccines and address justified medical skepticism within the Black community. Interventions to address COVID-19 vaccination in BYA in the South should focus on building feelings of trust in the COVID-19 vaccine through receiving information from trusted messengers, consistent and transparent messaging, and social support, with meaningful involvement from BYA in COVID-19 policies and programming for this age group.

## Abbreviations

BYA: Black or African American young adults
CDC: Centers for Disease Control and Prevention
FDA: Food and Drug Administration
NIMHD: National Institute on Minority Health and Health Disparities
SCT: social cognitive theory
